# Values in environmental research: Citizens’ views of scientists who acknowledge values

**DOI:** 10.1371/journal.pone.0186049

**Published:** 2017-10-25

**Authors:** Kevin C. Elliott, Aaron M. McCright, Summer Allen, Thomas Dietz

**Affiliations:** 1 Lyman Briggs College, Department of Fisheries and Wildlife, Department of Philosophy, Michigan State University, East Lansing, Michigan, United States of America; 2 Lyman Briggs College, Department of Sociology, Environmental Science and Policy Program, Michigan State University, East Lansing, Michigan, United States of America; 3 Department of Sociology, Michigan State University, East Lansing, Michigan, United States of America; 4 Department of Sociology, Environmental Science and Policy Program, Michigan State University, East Lansing, Michigan, United States of America; University of Missouri Columbia, UNITED STATES

## Abstract

Scientists who perform environmental research on policy-relevant topics face challenges when communicating about how values may have influenced their research. This study examines how citizens view scientists who publicly acknowledge values. Specifically, we investigate whether it matters: if citizens share or oppose a scientist’s values, if a scientist’s conclusions seem contrary to or consistent with the scientist’s values, and if a scientist is assessing the state of the science or making a policy recommendation. We conducted two 3x2 factorial design online experiments. Experiment 1 featured a hypothetical scientist assessing the state of the science on the public-health effects of exposure to Bisphenol A (BPA), and Experiment 2 featured a scientist making a policy recommendation on use of BPA. We manipulated whether or not the scientist expressed values and whether the scientist’s conclusion appeared contrary to or consistent with the scientist’s values, and we accounted for whether or not subjects’ values aligned with the scientist’s values. We analyzed our data with ordinary least squares (OLS) regression techniques. Our results provide at least preliminary evidence that acknowledging values may reduce the perceived credibility of scientists within the general public, but this effect differs depending on whether scientists and citizens share values, whether scientists draw conclusions that run contrary to their values, and whether scientists make policy recommendations.

## Introduction

Scientists frequently are asked to communicate with policy makers, journalists, and members of the general public on important environmental issues such as climate change, genetically engineered crops, food safety and nutrition, hydraulic fracturing, and pesticide safety. One of the major justifications for federal investment in scientific research is to provide the information our society needs to make well-informed decisions about these issues. Nevertheless, scientists face significant challenges when determining how to communicate effectively and ethically with different audiences. In particular, they must decide how best to maintain their objectivity while providing information that is relevant and accessible to their audiences [1–3].

One of the most significant challenges facing scientists who communicate with the public about major policy-relevant issues is how to address the potential roles that social, cultural, political, and economic values might play in their messages. Scholars distinguish these “non-epistemic” values from “epistemic” values (e.g., predictive accuracy, simplicity, explanatory power) employed within science to evaluate theory and validate knowledge [1]. Scientific objectivity and integrity often have been associated with insulating scientific reasoning from such non-epistemic values [4–7]. Moreover, while many members of the public accord considerable respect to scientists with regard to assessing facts, it is less clear that the public wishes to cede value judgments to scientists [8–10]. However, work in history, philosophy, and sociology of science suggests that non-epistemic value judgments often are intertwined with scientific research in ways that can be difficult to identify and eliminate [1, 11–14]. For example, such values can influence the specific questions asked about a research topic, the types of models and explanations regarded as most compelling and appropriate, the standards of evidence required for drawing conclusions, and the terminology used for describing findings [15–18]. For the remainder of this paper, we use “values” to refer to non-epistemic values.

Given the difficulty of eliminating values from scientific reasoning, some scholars suggest that the best course for scientists is to make their value commitments more transparent [3, 17, 19]. By doing so, they enable their audiences to better understand how such values might be influencing their reasoning, and they alleviate concerns that these values are surreptitiously influencing assessments billed as “value-free.” Recent efforts by many scholarly journals to require financial conflict-of-interest disclosures are one example of how value influences could be made more transparent. In addition, scientists could make deliberate attempts to acknowledge the ethical or social considerations (e.g., economic growth, public health, environmental protection) that they take to be particularly significant when addressing major policy-relevant issues. Even if scientists strive not to let these values influence their reasoning, their audiences still are better equipped to evaluate the scientists’ messages if they understand the values that could be influencing them.

## Research objectives

Proposals to promote greater transparency about scientists’ values raise significant empirical questions about how acknowledging values would affect public perceptions of scientists. Some scholars argue that scientists’ credibility could be eroded if they acknowledge their personal value commitments [6, 20]. For example, there is some evidence that when researchers disclose industry funding—which seems to reflect economic-growth values—it decreases perceived research quality and trust [21, 22]. This erosion of credibility may be especially likely among those citizens who do not share the scientists’ specific values or who do not respect the scientists’ research fields. For example, skepticism about scientists’ climate change findings among self-identified conservatives and Republicans in the US general public seems to be fueled at least in part by suspicions that such scientists are influenced by liberal or environmental values they oppose [23–25]. Further, self-identified conservatives report greater trust in scientists conducting research in the service of economic production (i.e., performing “production science”), while liberals place more trust in scientists examining the impacts of that economic production and new technologies (i.e., performing “impact science”) [26].

Other scholars suggest that scientists may increase their credibility by acknowledging their values, because members of the public might respect their efforts to be transparent and honest [17, 27]. This increased credibility may be more likely when citizens share the scientists’ specific values than when they do not [28]. It also may be more likely when scientists provide conclusions that appear divergent from their publicly stated values than when they offer ones that seem expected given their values. For instance, citizens may see scientists who offer conclusions *divergent from or contrary to* their values as more credible than those scientists who offer conclusions *convergent or consistent with* their values, because the former seem less likely than the latter to have been guided by motivated cognition [29].

Further, scientists’ credibility might be enhanced or degraded depending on whether the scientists acknowledge values when acting within or outside the conventional purview of science. For example, many hold the normative view that values are irrelevant and even corrupting to scientific reasoning itself, whereas values are necessary when formulating policy recommendations [30–32]. Therefore, citizens may be more willing to accept values statements from scientists acting at or beyond the boundaries of science (i.e., making policy recommendations) than from those acting clearly within those boundaries (i.e., assessing the state of the science) [8, 9].

Our study aims to examine the empirical support for these assertions about how citizens view scientists who publicly acknowledge their values. Briefly, we use the following research questions to frame our study of how a scientist’s public acknowledgement of values influences citizens’ positive affect toward and perceived trust in that scientist. To what extent does it matter:

- *if the citizens share or oppose the scientist’s values*?
- *if the scientist’s conclusion seems contrary to or consistent with the scientist’s values*?
- *if the scientist is assessing the state of the science or making a policy recommendation*?

We conducted two experiments to answer these research questions. Experiment 1 considers a scientist’s interpretation of the state of the science, while Experiment 2 considers a scientist’s policy recommendation. In each experiment, we manipulate whether a scientist’s conclusion appears contrary to or consistent with the scientist’s values, and we account for whether or not subjects’ values align with the scientist’s values. In particular, we instantiate values as broad policy preferences for either promoting economic growth or protecting public health.

Our experiments focus on the health risks of Bisphenol A (BPA), an endocrine-disrupting compound that has come under scrutiny because of its potential to interfere with hormonal activity in the bodies of humans and other species. BPA has been a common additive in plastics, including drinking bottles, medical devices, and the liners of food cans. Recent years have seen considerable scientific controversy about the potential health impacts of its use, with different studies coming to divergent conclusions [33–35]. In 2008, the U.S. Food and Drug Administration declared BPA to be safe, whereas the National Institute of Health’s National Toxicology Program expressed concerns about potential adverse health effects to humans from current exposure levels [36]. The ongoing controversy has been attributed to a number of factors, including differing perspectives on what kinds of biological effects count as “adverse,” efforts by the BPA industry to “manufacture” doubt about its adverse effects, and the hesitancy of regulatory agencies to take action solely on the basis of academic studies [34]. This is precisely the sort of debated, policy-relevant situation in which some scholars have called for scientists to be more transparent about their values [17].

## Experiment 1: Interpreting the state of the science

### Participants

We administered a survey-based experiment online via SurveyMonkey to participants recruited via Amazon Mechanical Turk (AMT), a crowdsourcing website where “requesters” solicit “workers” to perform “human intelligence tasks” (HITs) for pay. AMT has emerged as a practical way for recruiting a large number of participants from a reasonably wide cross-section of the general public for online experiments [37] or for testing new measurement instruments [38] across the social sciences [39–43].

To solicit a broad cross-section of research participants and minimize self-selection by AMT workers highly interested in BPA or public health, we advertised a HIT titled “Your Attitudes about Important Social Issues in the US.” We limited participation to adults residing in the United States. We paid participants $0.75 for completing the experiment, which took approximately six and a half minutes on average. Compared to a representative sample of the US general public, our AMT convenience sample is more male, younger, more highly educated, and more liberal. The sample contains 494 subjects who completed the entire experiment (of the 500 who began it) on September 17, 2015.

### The experiment

Our experiment employed a 3x2 full factorial design: values (values not mentioned, economic growth values present, or public health values present) by conclusion (BPA causes harm, BPA doesn’t cause harm). This was a fully randomized design that did not take into account any characteristics of the subjects. While in future research some stratification in the randomization process might be useful (e.g., to balance sample size across some of our key non-experimental independent variables), we had no a priori information that would guide a more complex design of this sort. The exact stimulus messages presented in each of the six resulting conditions are presented in Part A of S1 File. In our analyses we divided subjects into those who favored promoting economic growth over protecting public health and those who favored protecting public health over promoting economic growth and then examined the effects of our experimental manipulations within those groups.

The Social Science Behavioral/Education Institutional Review Board (SIRB) of the Human Research Protection Program at Michigan State University approved this research (IRB#x13-556e) (Exempt 2). After reading a consent page and agreeing to participate in our study, subjects answered questions about their preference for promoting economic growth or protecting public health and about whether they view certain behaviors by scientists as ethical or unethical. They then were randomly assigned to one of the six conditions above. All subjects received the following background information before reading their brief experimental message:

For several decades, a scientist named Dr. Riley Spence has been doing research on chemicals used in consumer products. One chemical—Bisphenol A, popularly known as BPA—is found in a large number of consumer products and is suspected of posing risks to human health. Yet, scientists do not agree about these possible health risks. Dr. Spence recently gave a public talk in Washington, D.C. about BPA research. Here is the final slide from his presentation.

After reading their assigned stimulus message, subjects answered two open-ended comprehension questions and two closed-ended manipulation check questions. Subjects then answered two sets of questions about their perceptions of Dr. Spence. After subjects answered a few brief demographic, social, and political questions, we thanked them for their participation and debriefed them about our research question.

### Variables

We employed two composite outcome variables in our analyses. *Positive affect* (Cronbach’s alpha = 0.96) is a semantic differential scale that is the average of responses to seven items, each of which was measured on a seven-point scale. It measures the positive affect that subjects have toward Dr. Spence. The seven items, whose response categories ranged from “not at all” = 1 to “very” = 7 were: competent, credible, expert, honest, intelligent, sincere, and trustworthy. *Perceived trust* (Cronbach’s alpha = 0.98) is a scale that is the average of seven measures of perceived trust, each of which was measured on a seven-point scale (“completely distrust” = 1 to “completely trust” = 7) using the following items: draw scientific conclusions that are independent from his research funders; draw scientific conclusions that are independent from his own political values; fully consider other scientific studies with results that don’t match his political values; fairly report all sides of BPA research to the news media; fairly report all sides of BPA research to Congress; clearly represent uncertainties about the effects of BPA to the news media; and clearly represent uncertainties about the effects of BPA to Congress.

We used three dummy variables to account for our experimental conditions. Two dummy variables distinguish whether the scientist was labeled as favoring economic growth (*scientist values economic growth*) or as favoring protecting public health (*scientist values public health*) with “no values present” as the reference category. A third dummy variable differentiates the condition where the scientist concludes that BPA causes harm (*scientist concludes BPA causes harm*) from the one where the “scientist concludes BPA does not cause harm,” which serves as the reference category.

We assessed subjects’ pre-existing preference for either promoting economic growth or protecting public health with the following question: “National policies are often the result of trade-offs between important priorities. When there is a tradeoff between promoting economic growth and protecting public health, which BEST captures your preference?” The four response categories (“strong preference for promoting economic growth,” “mild preference for promoting economic growth,” “mild preference for protecting public health,” and “strong preference for protecting public health”) were dichotomized to represent a preference for either promoting economic growth (37.1% of subjects) or protecting public health (62.9% of subjects).

We also employed four demographic, social, and political variables as statistical controls in our analyses. *Female* is a dummy variable for gender: “male” = 0 and “female” = 1. Approximately 52% of our subjects are male. *Age* is measured with ten categories: “18–19” = 1 to “60 or higher” = 10. Approximately 75% of our subjects are younger than 40 years old. *Education* is measured by the highest degree earned: “up to high school diploma or equivalent” = 1 to “graduate/professional degree” = 4. Approximately 53% of our subjects have a bachelor’s degree. *Political ideology* is measured on a 7-point scale from “very conservative” = 1 to “very liberal” = 7, with “middle-of-the-road” = 4. Approximately 52% of our subjects identify as slightly to very liberal. We treat age, education and political ideology as continuous variables to preserve degrees of freedom. Thus, our analysis may not capture any strongly non-linear effects of these variables.

### Analytical techniques

We analyzed our data with a series of ordinary least squares (OLS) regression models using the Stata 14.1 software package. We also estimated Huber-White standard errors that are robust with regard to non-normality and heteroscedasticity; those estimates yielded substantively identical results and are not reported here. Since the positive affect scale and the perceived trust scale each take on a large number of distinct values (64 and 43, respectively), we felt justified in treating these as continuous outcome variables in OLS regression. All of these models included our four demographic, social, and political variables as controls. For each outcome variable (*positive affect* and *perceived trust*), we ran a model examining the main effects of the following four predictor variables: *scientist values economic growth* and *scientist values public health* with “no values present” as the reference category; *scientist concludes BPA causes harm* with “scientist concludes BPA does not cause harm” as the reference category; and *subject favors public health* with “subject favors economic growth” as the reference category.

To examine those interaction effects suggested in our research questions, we ran OLS regression models predicting each outcome variable with the two values statements dummy variables (*scientist values economic growth* and *scientist values public health*) within four subsamples. We defined these four subsamples by the scientist’s conclusion about the state of the science on BPA (whether the scientist concludes BPA causes harm or concludes BPA does not cause harm) and the subject’s own value preference (whether the subject favors promoting economic growth versus favors protecting public health). Running separate analyses within subgroups allows each subgroup to have its own estimated error variance but reduces degrees of freedom for each analysis. An alternative suggested by a reviewer is to use all cases to preserve degrees of freedom, and thus power, using interaction effects to allow coefficients to vary across subgroups. However, this approach constrains estimated error variances to be equal across subgroups and introduces high collinearity among the interaction terms. We also have conducted the analysis using interaction effects and all cases, and the results are essentially identical to those reported here.

We report p values as is standard practice. In addition to the standard cautions on the use of p values [44], we emphasize the following. Since we are using a convenience sample, while p has the usual interpretation for the experimentally manipulated variables, the p value for other variables is best interpreted as a comparison to a purely random variable. As suggested by a reviewer, we also note that the power of statistical tests in the subgroups is less than ideal. We emphasize again that our results should be viewed as exploratory and in need of replication.

### Results

**Fig 1:**
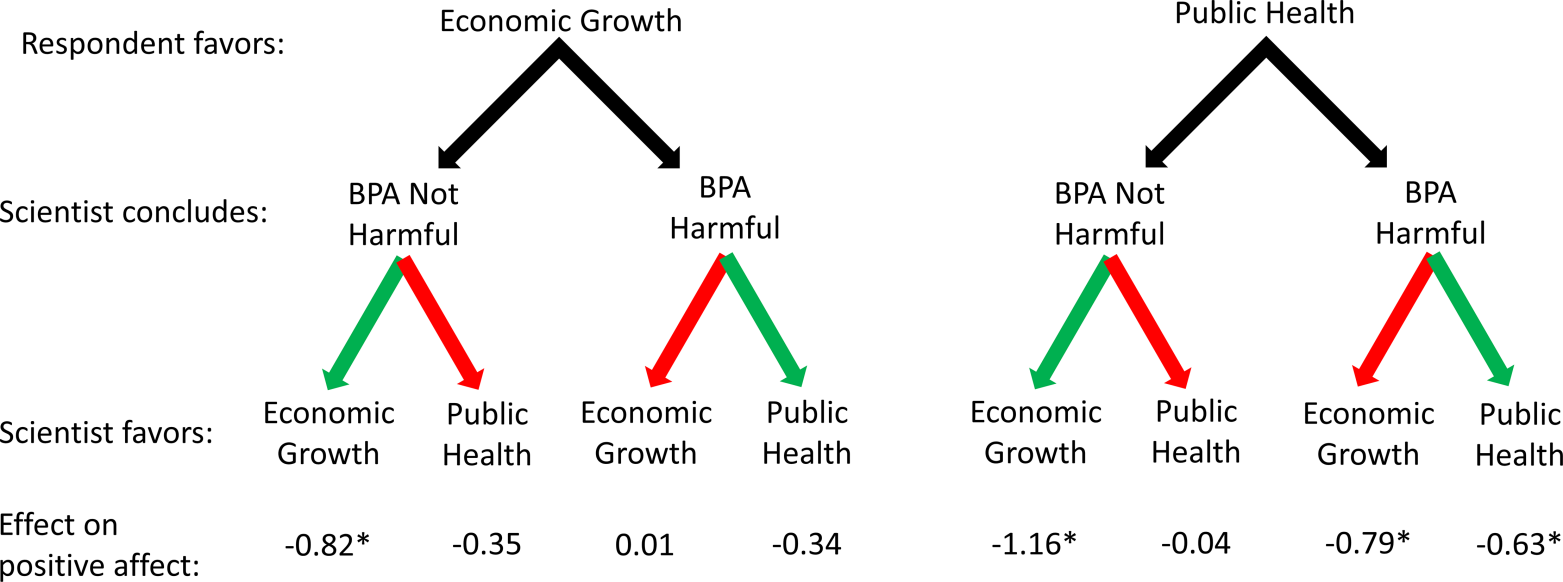
Effects of scientists’ conclusions and expressed values on respondents’ positive affect toward them. Effects are relative to a scientist not expressing a preference for particular values and are net of control variables; see Table 1. Green arrows are used when the scientist’s conclusion coincides with the scientist’s values; red arrows are used when the scientist’s conclusion conflicts with the scientist’s values.

**Fig 2:**
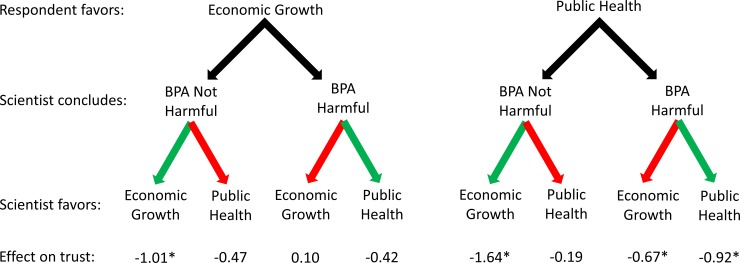
Effects of scientists’ conclusions and expressed values on respondents’ trust in them. Effects are relative to a scientist not expressing a preference for particular values and are net of control variables; see Table 1. Green arrows are used when the scientist’s conclusion coincides with the scientist’s values; red arrows are used when the scientist’s conclusion conflicts with the scientist’s values.

Tables 1 and 2 present the results of OLS regression models explaining positive affect toward and perceived trust in the scientist who offered an interpretation of the state of the science on BPA, respectively. In each table, the first model estimates main effects in the full sample and the four subsequent models estimate the effects of the two values dummy variables in each of four subsamples. Figs 1 and 2 visually represent selected results from the tables.

**Table 1:** Measures of the effect of scientists’ conclusions and expressed values on respondents’ positive attitude toward them (Experiment 1).

	Overall	Subject Favors Economic Growth		Subject Favors Public Health	
Predictors		Scientist Concludes BPA Does Not Cause Harm	Scientist Concludes BPA Causes Harm	Scientist Concludes BPA Does Not Cause Harm	Scientist Concludes BPA Causes Harm
Scientist Values Economic Growth	-0.75* (0.15)	-0.82* (0.40)	0.01 (0.29)	-1.16* (0.26)	-0.79* (0.25)
Scientist Values Public Health	-0.31* (0.15)	-0.35 (0.37)	-0.34 (0.29)	-0.04 (0.28)	-0.63* (0.25)
Scientist Concludes BPA Causes Harm	1.05* (0.12)				
Subject Favors Public Health	-0.10 (0.13)				
Female	-0.04 (0.12)	-0.35 (0.32)	0.17 (0.24)	0.06 (0.23)	0.00 (0.20)
Age	0.06* (0.03)	0.15 (0.09)	0.27* (0.06)	-0.07 (0.05)	0.08 (0.05)
Education	-0.10 (0.06)	-0.03 (0.14)	0.10 (0.12)	-0.38* (0.11)	-0.01 (0.10)
Political Ideology (Conservative to Liberal)	0.01 (0.04)	-0.12 (0.10)	0.20* (0.07)	-0.08 (0.07)	0.06 (0.06)
Constant	4.89* (0.29)	5.34* (0.84)	3.29* (0.54)	6.35* (0.55)	5.58* (0.47)
Observations	492	90	94	165	143
Adjusted R^2^	0.18	0.14	0.25	0.19	0.10

Notes: Standard errors in parentheses.

*p<0.05

**Table 2:** Measures of the effect of scientists’ conclusions and expressed values on respondents’ trust (Experiment 1).

	Overall	Subject Favors Economic Growth		Subject Favors Public Health	
Predictors		Scientist Concludes BPA Does Not Cause Harm	Scientist Concludes BPA Causes Harm	Scientist Concludes BPA Does Not Cause Harm	Scientist Concludes BPA Causes Harm
Scientist Values Economic Growth	-0.89* (0.18)	-1.01* (0.50)	0.10 (0.32)	-1.64* (0.30)	-0.67* (0.29)
Scientist Values Public Health	-0.46* (0.18)	-0.47 (0.46)	-0.42 (0.32)	-0.19 (0.32)	-0.92* (0.30)
Scientist Concludes BPA Causes Harm	1.33* (0.15)				
Subject Favors Public Health	-0.22 (0.16)				
Female	-0.06 (0.15)	-0.81* (0.40)	0.15 (0.26)	0.21 (0.26)	0.08 (0.23)
Age	0.00 (0.03)	0.15 (0.11)	0.23* (0.07)	-0.17* (0.06)	0.04 (0.05)
Education	-0.03 (0.07)	0.04 (0.18)	0.11 (0.12)	-0.22 (0.13)	0.03 (0.12)
Political Ideology (Conservative to Liberal)	0.00 (0.05)	-0.05 (0.12)	0.25* (0.08)	-0.16 (0.08)	0.05 (0.08)
Constant	4.66* (0.35)	4.76* (1.05)	3.07* (0.59)	6.41* (0.64)	5.43* (0.56)
Observations	492	90	94	165	143
Adjusted R^2^	0.19	0.12	0.22	0.24	0.08

Notes: Standard errors in parentheses.

*p<0.05

In the two overall regression models with only additive effects, subjects exposed to a scientist mentioning either economic growth values or public health values report lesser positive affect toward and perceived trust in the scientist than do subjects exposed to a scientist mentioning no values. The effect of the presence of economic growth values is substantially greater than the effect of the presence of public health values both on positive affect (F_1,483_ = 9.01, p = 0.003) and on perceived trust (F_1,483_ = 5.95, p = 0.015). Also, subjects exposed to a scientist who concludes that BPA causes harm report greater positive affect toward and perceived trust in the scientist than do subjects exposed to a scientist who concludes that BPA does not cause harm. Subjects’ own values (i.e., protecting public health versus promoting economic growth) have no effect on either positive affect or perceived trust. Also, age is the only social, demographic, or political control variable that has a statistically significant effect on either outcome variable in the overall sample, with older subjects reporting greater positive affect toward the scientist than their younger counterparts. At the suggestion of a reviewer, we also re-estimated this model without controlling for political ideology and found no substantial changes in our results.

Shifting to the results of the models with the four subsamples allows us to explore the more nuanced patterns suggested by the scholars’ views mentioned earlier. Exposure to a scientist acknowledging economic growth values significantly decreases positive affect toward and perceived trust in that scientist for subjects in three of four subsamples. The exception is the group of subjects who favor economic growth over public health and who were exposed to a scientist concluding that BPA causes harm. Exposure to a scientist acknowledging public health values significantly decreases positive affect toward and perceived trust in that scientist only for subjects who favor public health over economic growth and who were exposed to a scientist who concludes that BPA causes harm.

## Experiment 2: Making a policy recommendation

### Participants

As with Experiment 1, we administered a survey-based experiment online via SurveyMonkey to subjects recruited via AMT using a HIT titled “Your Attitudes about Important Social Issues in the US.” We again limited participation to adults residing in the United States and paid participants $0.75 for completing the experiment, which took approximately six and a half minutes on average. We also excluded any Experiment 1 participants from Experiment 2. Compared to a representative sample of the US general public, our AMT convenience sample is more male, younger, more highly educated, and more liberal. The sample contains 482 subjects who completed the entire experiment (of the 500 who began it) on September 18, 2015.

#### The experiment

While Experiment 1 focused on a scientist mentioning or not mentioning values when interpreting the science, Experiment 2 focused on a scientist acknowledging or not acknowledging values when making a policy recommendation. This experiment employed a 3x2 full factorial design: values (values not mentioned, economic growth values present, or public health values present) by conclusion (regulate BPA more strongly, reduce regulation of BPA). Again, this was a fully randomized design with no stratification. The exact stimulus messages presented in each of the six resulting six conditions are presented in Part B of S1 File. Other than the difference in experimental stimuli, the design and order of Experiment 2 was identical to that of Experiment 1.

### Variables

We employed the same two composite outcome variables as in Experiment 1: *positive affect* (Cronbach’s alpha = 0.96) and *perceived trust* (Cronbach’s alpha = 0.98). We used three dummy variables to account for our experimental conditions. The first two are the same as in Experiment 1: *scientist values economic growth* and *scientist values public health* with “no values present” as the reference category. A third dummy variable differentiates the condition where the scientist recommends we regulate BPA more strongly (*scientist calls for stronger regulation*) from the one where the “scientist calls for reducing regulation,” which serves as the reference category. We further account for subjects’ pre-existing preference for either promoting economic growth (31.3% of subjects) or protecting public health (69.0% of subjects). We also employ the same four demographic, social, and political variables as statistical controls as in Experiment 1. Approximately 51% of our subjects are male, 75% of our subjects are younger than 40 years old, 53% of our subjects have a bachelor’s degree, and 55% of our subjects identify as slightly to very liberal. As in Experiment 1, we treat age, education, and political ideology as continuous variables to preserve degrees of freedom. Thus, our analysis may not capture any strongly non-linear effects of these variables.

### Analytical techniques

We employed the same analytical techniques using Stata 14.1 as we did in Experiment 1. We analyzed our data with a series of OLS regression models, each of which included our four demographic, social, and political variables as controls. We also estimated Huber-White standard errors that are robust with regard to non-normality and heteroscedasticity; those estimates yielded substantively identical results and are not reported here. Again, OLS is justified because our two outcome variables take on a large number of distinct values: 58 for positive affect and 43 for perceived trust. For each outcome variable (*positive affect* and *perceived trust*), we ran a model examining the main effects of the following four predictor variables: *scientist values economic growth* and *scientist values public health* with “no values present” as the reference category; *scientist calls for stronger regulation* with “scientist calls for reduced regulation” as the reference category; and *subject favors public health* with “subject favors economic growth” as the reference category. To examine those interaction effects suggested in our research questions, we ran OLS regression models predicting each outcome variable with the two values statements dummy variables (*scientist values economic growth* and *scientist values public health*) within our four subsamples.

### Results

**Fig 3:**
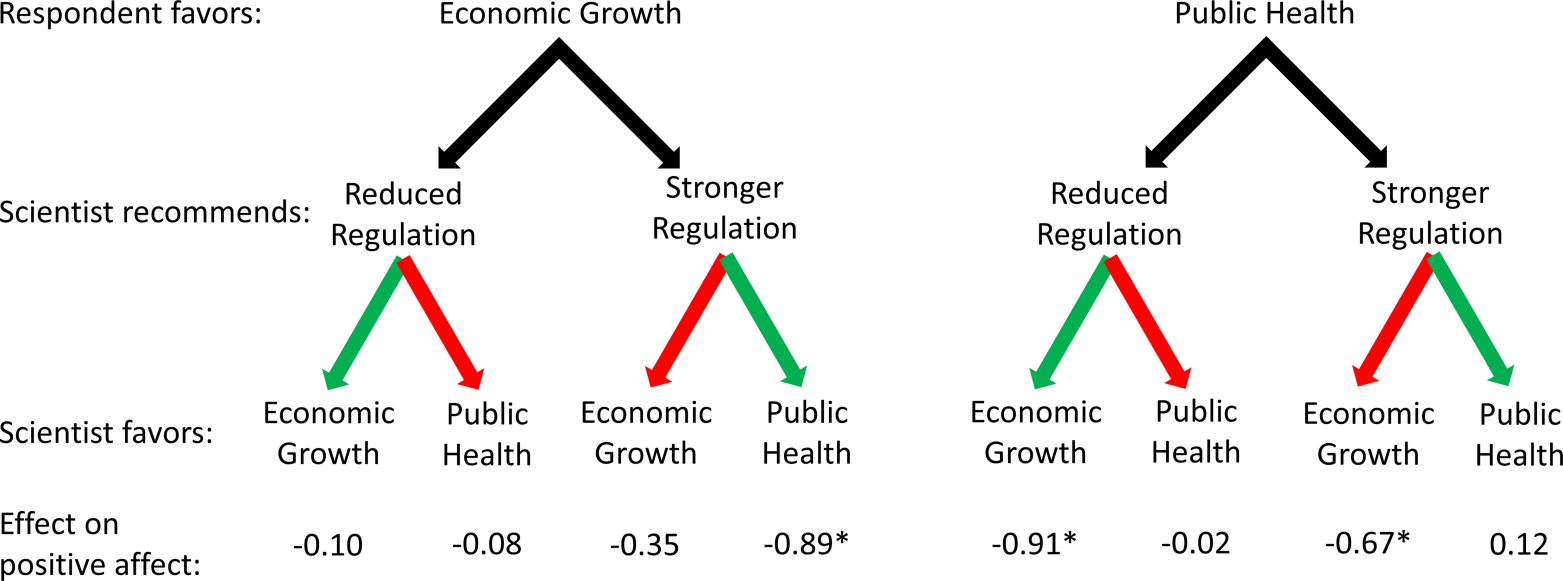
Effects of scientists’ policy recommendations and expressed values on respondents’ positive affect toward them. Effects are relative to a scientist not expressing a preference for particular values and are net of control variables; see Table 1. Green arrows are used when the scientist’s recommendation coincides with the scientist’s values; red arrows are used when the scientist’s recommendation conflicts with the scientist’s values.

**Fig 4:**
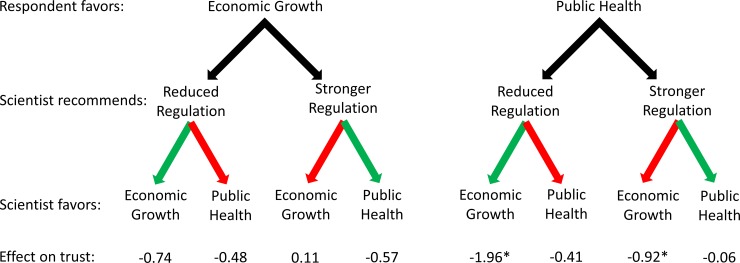
Effects of scientists’ policy recommendations and expressed values on respondents’ trust in them. Effects are relative to a scientist not expressing a preference for particular values and are net of control variables; see Table 1. Green arrows are used when the scientist’s recommendation coincides with the scientist’s values; red arrows are used when the scientist’s recommendation conflicts with the scientist’s values.

Tables 3 and 4 present the results of OLS regression models explaining positive affect toward and perceived trust in the scientist who made a policy recommendation about BPA, respectively. None of our social, demographic, and political control variables have a statistically significant effect on either positive affect or perceived trust in our overall sample. Figs 3 and 4 visually represent selected results from the tables.

**Table 3:** Measures of the effect of scientists’ policy recommendations and expressed values on respondents’ positive attitude toward them (Experiment 2).

	Overall	Subject Favors Economic Growth		Subject Favors Public Health	
Predictors		Scientist Concludes BPA Does Not Cause Harm	Scientist Concludes BPA Causes Harm	Scientist Concludes BPA Does Not Cause Harm	Scientist Concludes BPA Causes Harm
Scientist Values Economic Growth	-0.55* (0.16)	-0.10 (0.47)	-0.35 (0.42)	-0.91* (0.31)	-0.67* (0.20)
Scientist Values Public Health	-0.02 (0.15)	-0.08 (0.41)	-0.89* (0.43)	-0.02 (0.29)	0.12 (0.18)
Scientist Concludes BPA Causes Harm	1.24* (0.12)				
Subject Favors Public Health	-0.06 (0.14)				
Female	0.10 (0.13)	-0.30 (0.36)	0.39 (0.36)	-0.35 (0.23)	0.58* (0.15)
Age	0.02 (0.03)	-0.12 (0.09)	0.16 (0.10)	0.00 (0.06)	0.06 (0.03)
Education	-0.10 (0.06)	-0.14 (0.19)	0.01 (0.19)	-0.23 (0.12)	-0.03 (0.07)
Political Ideology (Conservative to Liberal)	-0.03 (0.04)	-0.07 (0.12)	0.20 (0.10)	-0.06 (0.08)	-0.09 (0.05)
Constant	4.89* (0.29)	6.09* (0.80)	4.19* (0.75)	5.63* (0.62)	5.90* (0.38)
Observations	471	79	67	154	171
Adjusted R^2^	0.20	0.06	0.17	0.12	0.20

Notes: Standard errors in parentheses.

*p<0.05

**Table 4:** Measures of the effect of scientists’ policy recommendations and expressed values on respondents’ trust (Experiment 2).

	Overall	Subject Favors Economic Growth		Subject Favors Public Health	
Predictors		Scientist Concludes BPA Does Not Cause Harm	Scientist Concludes BPA Causes Harm	Scientist Concludes BPA Does Not Cause Harm	Scientist Concludes BPA Causes Harm
Scientist Values Economic Growth	-1.05* (0.19)	-0.74 (0.57)	0.11 (0.42)	-1.96* (0.36)	-0.92* (0.26)
Scientist Values Public Health	-0.27 (0.17)	-0.48 (0.49)	-0.57 (0.43)	-0.41 (0.33)	-0.06 (0.23)
Scientist Concludes BPA Causes Harm	1.46* (0.15)				
Subject Favors Public Health	-0.22 (0.17)				
Female	0.12 (0.15)	-0.44 (0.44)	0.08 (0.36)	-0.15 (0.27)	0.68* (0.20)
Age	0.00 (0.03)	-0.10 (0.10)	0.06 (0.10)	-0.00 (0.06)	0.06 (0.04)
Education	-0.11 (0.07)	-0.23 (0.23)	0.23 (0.19)	-0.15 (0.14)	-0.15 (0.09)
Political Ideology (Conservative to Liberal)	0.02 (0.05)	0.07 (0.14)	0.10 (0.10)	0.04 (0.09)	0.11 (0.07)
Constant	4.59* (0.34)	5.57* (0.96)	4.13* (0.75)	4.72* (0.72)	6.01* (0.49)
Observations	471	79	67	154	171
Adjusted R^2^	0.22	0.06	0.10	0.20	0.17

Notes: Standard errors in parentheses.

*p<0.05

In the two overall regression models with only additive effects, subjects exposed to a scientist mentioning economic growth values report lesser positive affect toward and perceived trust in the scientist than do subjects exposed to a scientist mentioning no values. Exposure to a scientist mentioning public health values has no effect on subjects’ perceptions of the scientist. Also, while subjects’ own values have no effect on such perceptions, subjects exposed to a scientist who calls for more strongly regulating BPA report greater positive affect toward and perceived trust in the scientist than do subjects exposed to a scientist who calls for reducing regulation of BPA. At the suggestion of a reviewer, we also re-estimated this model without controlling for political ideology and found no substantial changes in our results.

We now shift to the results of the models using data from the four subsamples. Exposure to a scientist acknowledging economic growth values significantly decreases positive affect toward and perceived trust in that scientist, but only among subjects who favor public health over economic growth. Whether this pattern appears or not is not affected by the scientist’s policy recommendation. Exposure to a scientist acknowledging public health values significantly decreases positive affect toward a scientist only for subjects who favor economic growth over public health and who were exposed to a scientist recommending that BPA be more strongly regulated. Among all other subsamples, exposure to a scientist mentioning public health values has no statistically significant effect on either positive affect toward or perceived trust in that scientist.

## Discussion

In response to the ways in which values intertwine with scientific research, some scholars argue that scientists should make their value commitments more transparent [3, 17, 19]. Such calls for greater transparency about scientists’ values raise important empirical questions about how scientists’ acknowledgements of values may affect public perceptions of them. Focusing on the topic of the health risks of BPA, we conducted two experiments to provide initial answers to three key questions. Briefly, when a scientist publicly acknowledges values, how do the following matter for how citizens view that scientist:

- *if the citizens share or oppose the scientist’s values*?
- *if the scientist’s conclusions seem contrary to or consistent with the scientist’s values*?
- *if the scientist is assessing the state of the science or making a policy recommendation*?

Box 1 below displays our major findings, which we summarize here. No subsample in either experiment—not even those where the subjects shared the scientist’s values—saw an increase in subjects’ positive affect toward or perceived trust in a scientist acknowledging values. This offers *no support* for the perspective that scientists’ credibility may be enhanced by mentioning their values [17, 27]. Acknowledging values either decreased the scientist’s credibility or left it unchanged, depending on the circumstances.

**Box 1: Summary of major findings.**

1. No context, not even when laypeople share the scientist’s values, sees a statistically significant increase in positive affect toward or perceived trust in a scientist who acknowledges values. Depending on the circumstances, acknowledging values either decreases or does not affect the scientist’s credibility.
2. Compared to acknowledging values associated with public health, acknowledging values associated with economic growth tends to decrease positive affect and perceived trust.
3. Compared to concluding that a potential threat causes no harm or needs no more regulation, concluding that a potential threat does cause harm or requires more regulation increases positive affect and perceived trust.
4. Compared to acknowledging values shared by laypeople, acknowledging values that are not shared tends to decrease positive affect and perceived trust.
5. Even when acknowledging values shared by laypeople, positive affect and perceived trust are decreased when scientists report scientific conclusions and policy recommendations consistent with their values.
6. Positive affect and perceived trust are often not reduced when scientists report scientific conclusions and policy recommendations that run counter to their acknowledged values.
7. Acknowledging values when drawing scientific conclusions tends to decrease positive affect and perceived trust.
8. The decrease in positive affect and perceived trust when acknowledging values when making policy recommendations is less straightforward; it depends on the values espoused by the scientist and by laypeople.

### Shared or opposing values

Our analyses indicate that acknowledging values is more likely to decrease a scientist’s credibility when citizens do not share the scientist’s values than when they do. Our results provide *strong support* for this claim when subjects value public health and the scientist values economic growth. Regardless of whether the scientist was assessing the state of the science or making a policy recommendation and regardless of the nature of the scientist’s conclusion in either experiment, positive affect toward and perceived trust in the scientist was reduced. Yet, this claim only receives *weak support* when subjects value economic growth and the scientist values public health. In this context, only positive affect is reduced, and this occurs only in Experiment 2 when the scientist calls for stronger regulation of BPA (a situation where the scientist’s recommendation appears consistent with the scientist’s values).

Our results also indicate that shared values between scientists and citizens are not always sufficient to preserve a scientist’s credibility. While the credibility of a scientist acknowledging a value shared by subjects was unaffected when making a policy recommendation (Experiment 2), this was not the case when the scientist was assessing the state of the science (Experiment 1). In this latter context, the scientist’s credibility was reduced in half of the situations—every time when the scientist’s conclusion seemed consistent with the scientist’s values, but not once when the conclusion seemed contrary to the acknowledged values. We now shift to focus more directly on this relationship between values and conclusions.

### Conclusions contrary to or consistent with values

Our analyses indicate that citizens may be more skeptical of scientists whose conclusions seem to follow from their values than of scientists whose conclusions seem contrary to their values, likely because of the concern that the latter may have reached their potentially biased conclusions via motivated cognition [45]. The results from Experiment 1 offer *moderate support* for the claim that scientists’ credibility is reduced when they offer conclusions that seem convergent with their reported values. Positive affect toward and perceived trust in the scientist are reduced in three of the four subsamples (all but when subjects valuing economic growth evaluate a scientist who values public health and concludes that BPA causes harm). Yet, when scientists offer conclusions that seem divergent from their reported values, positive affect and perceived trust are reduced in only one of the four subsamples (when subjects valuing public health evaluate a scientist who values economic growth and concludes that BPA causes harm).

Experiment 2 provides only *weak support* for the assertion that scientists’ credibility depends upon whether their decisions seem convergent with or divergent from their reported values. When scientists offer conclusions that seem convergent with their reported values, both positive affect and perceived trust are reduced in only one subsample: when subjects valuing public health evaluate a scientist valuing economic growth who calls for reducing regulation of BPA. Positive affect (but not perceived trust) is reduced in another subsample: when subjects valuing economic growth valuate a scientist valuing public health who calls for stronger regulation. Paralleling what we found in Experiment 1, when scientists offer conclusions that seem divergent from their reported values, positive affect and perceived trust are reduced in only one of the four subsamples (when subjects valuing public health evaluate a scientist who values economic growth and calls for stronger regulation).

### Assessing the science or making a policy recommendation

Because values are commonly recognized as being relevant to policy-making but not to scientific reasoning [30–32], citizens may have fewer concerns about values statements when scientists make policy recommendations than when they assess the state of the science [8, 9]. Our results provide *moderate support* for this assertion, at least in those circumstances when there is alignment between the scientist’s and subjects’ values. In this situation, both positive affect toward and perceived trust in a scientist assessing the state of the science are reduced when the scientist offered a conclusion contrary to the scientist’s values (Experiment 1). Yet, when the scientist made a policy recommendation in this same context, positive affect and perceived trust are unaffected. Thus, in some circumstances, the credibility of a scientist acknowledging values is affected by whether the scientist is assessing the state of the science or is making a policy recommendation.

### Recommendations for further research

While our findings are compelling, they nevertheless result from a single study on a single public policy issue in which we analyzed data from two convenience samples. Further work is clearly warranted in what we anticipate will be a growing literature. This is especially the case as scientists more frequently communicate with laypeople and policy-makers about policy-relevant scientific issues via social media. Given this, we end with a few recommendations for moving this scholarship forward.

Additional research should aim to replicate and extend this study to more completely assess the consistency and generalizability of its results. While our two experiments focused on one specific research topic (i.e., potential health risks of BPA), future research should explore a wider array of substantive areas (e.g., agrifood products, pharmaceutical drugs or dietary supplements, energy development, ecological change). This is important, since citizens may be willing to accept the influence of values in some areas of science but not in others.

Future work may further explore nuanced patterns like those we found here by employing other scholarly conceptions of science: e.g., “frontier science” versus “core science” [46] or Funtowicz and Ravetz’s “post-normal science” versus “applied science” [47]. For instance, earlier we mentioned Schnaiberg’s distinction between science in the service of economic production (i.e., “production science”) and science examining the impacts of that economic production or of new technologies (i.e., “impact science”) [48]. This conceptualization helps explain support for or opposition to fields of policy-relevant science [26], and it also may be efficacious for helping us understand the likely nuanced dynamics of values and credibility.

Finally, since earlier work argues that conservative Republicans’ skepticism of climate science seems at least partially due to concerns that climate scientists are influenced by liberal or environmental values they oppose [23–25], the emerging literature on public skepticism of science [49–52] and further work on the roles of values in science [11, 15, 53, 54] may mutually benefit from cross-fertilization. It does seem reasonable that some of the public skepticism of other policy-relevant areas of science (e.g., the efficacy and safety of childhood vaccines, the safety of nuclear power facilities, the health impacts of genetically modified organisms) is driven by citizens’ concerns that the scientists performing this research do not share their values. Thus, future research may improve our understanding of how values influence public skepticism about policy-relevant areas of science.

## Conclusion

The results of both experiments suggest that publicly acknowledging values may diminish the perceived credibility of scientists within the general public. Yet, the patterns of our results were rather nuanced. That is, we found at least some evidence that citizens’ views of scientists depend upon whether (a) the citizens share or oppose the scientists’ values, (b) the scientists’ conclusions seem contrary to or consistent with the scientists’ values, and (c) the scientists are assessing the state of the science or are making a policy recommendation.

Further research is needed to establish the consistency and generalizability of these results across other topics related to environmental research. Moreover, these empirical findings are just one of many factors that scientists need to take into account when deciding how to present policy-relevant findings to the public. For example, even if acknowledging values diminishes their credibility, scientists may have ethical responsibilities to be transparent about the ways in which values could have influenced their research or their policy recommendations [17, 19]. Nevertheless, these results provide important information for scientists who perform research on policy-relevant topics related to environmental issues. In particular, they should be prepared that acknowledging values may have the consequence of diminishing their credibility, and they should consider whether additional steps are warranted to address this consequence.

## Supporting information

S1 File Supplementary material. (DOCX)
