# Supplementary material for: Values in environmental research: Citizens’ views of scientists who acknowledge values
Source: PLoS One. 2017 Oct 25;12(10):e0186049. doi: 10.1371/journal.pone.0186049 (PMC5656313; doi:10.1371/journal.pone.0186049)

**Part A. Experiment 1 Messages**

Condition 1: scientist does not mention values; scientist concludes that BPA causes harm


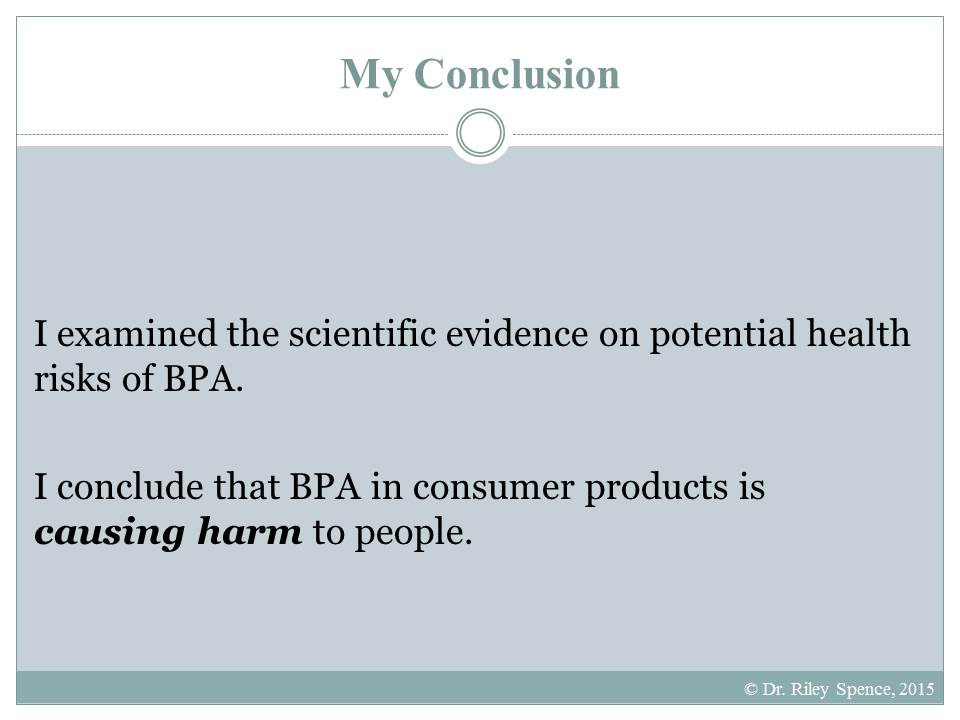


Condition 2: scientist does not mention values; scientist concludes that BPA doesn’t cause harm


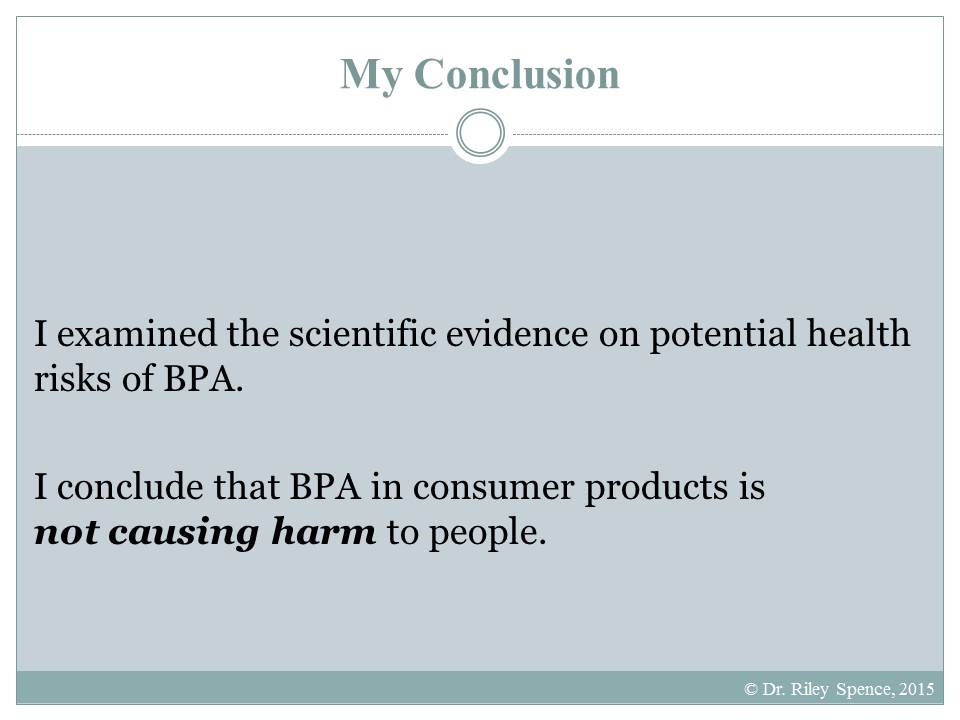


Condition 3: scientist mentions public health values; scientist concludes that BPA causes harm


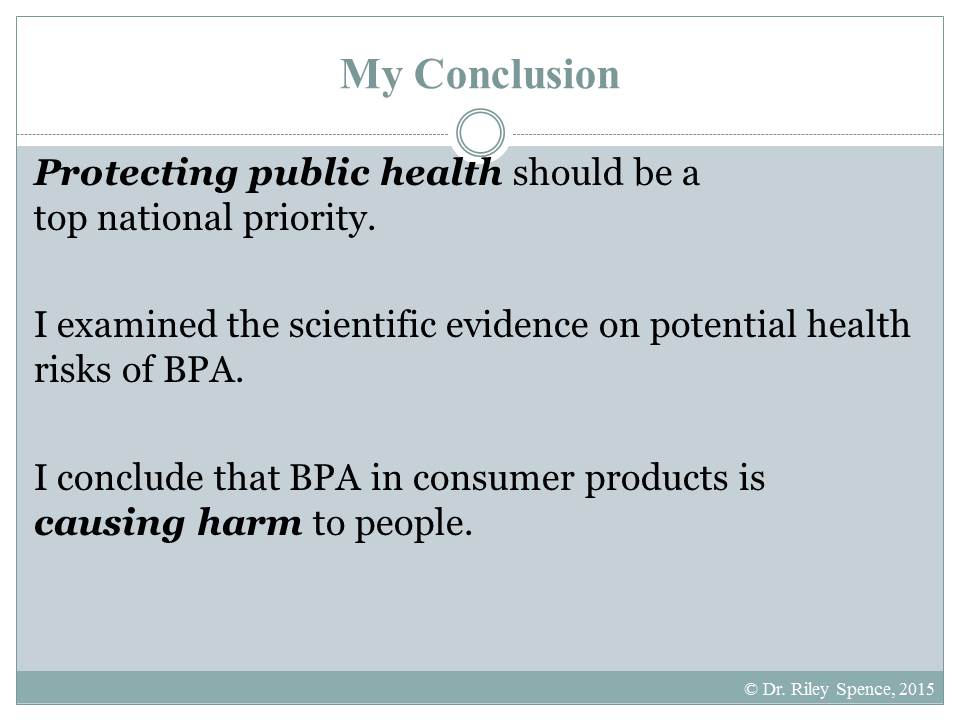


Condition 4: scientist mentions public health values; scientist concludes that BPA doesn’t cause harm


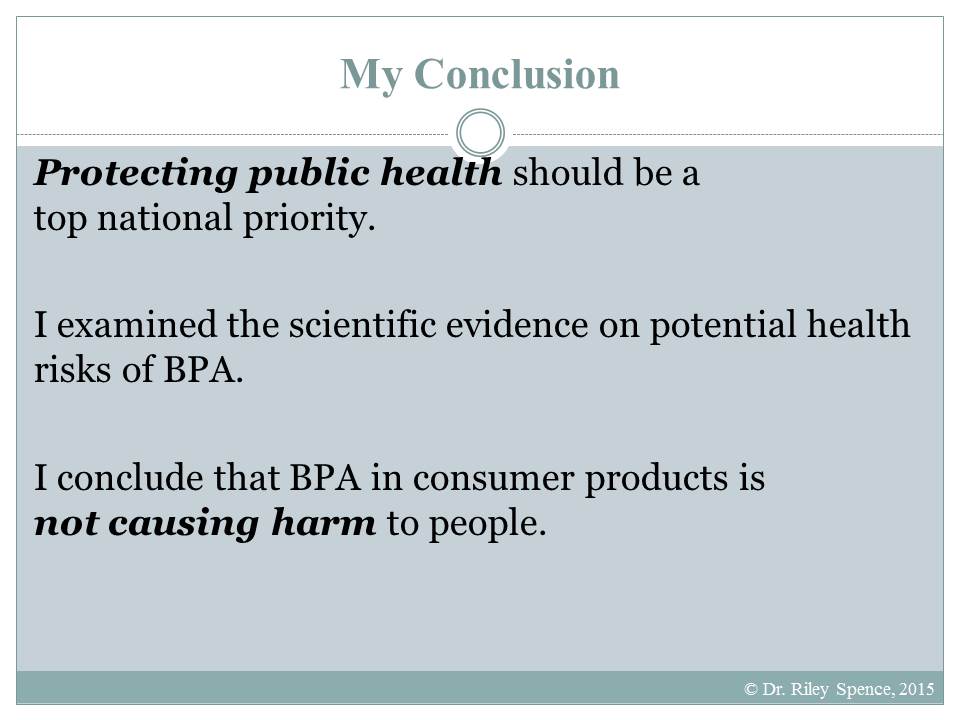


Condition 5: scientist mentions economic growth values; scientist concludes that BPA causes harm


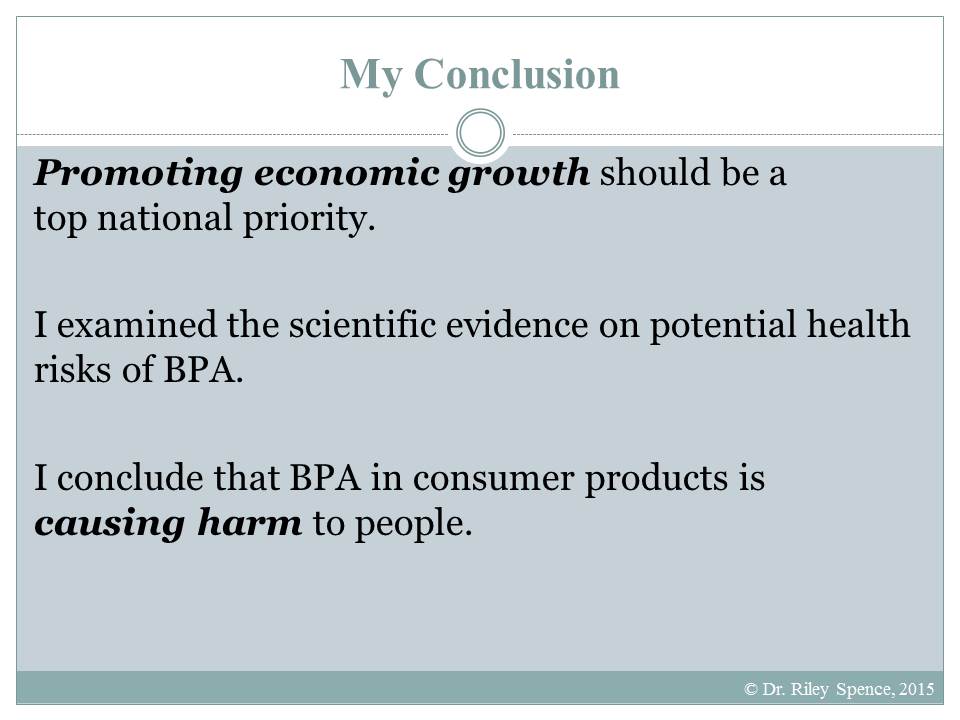


Condition 6: scientist mentions economic growth values; scientist concludes that BPA doesn’t cause harm


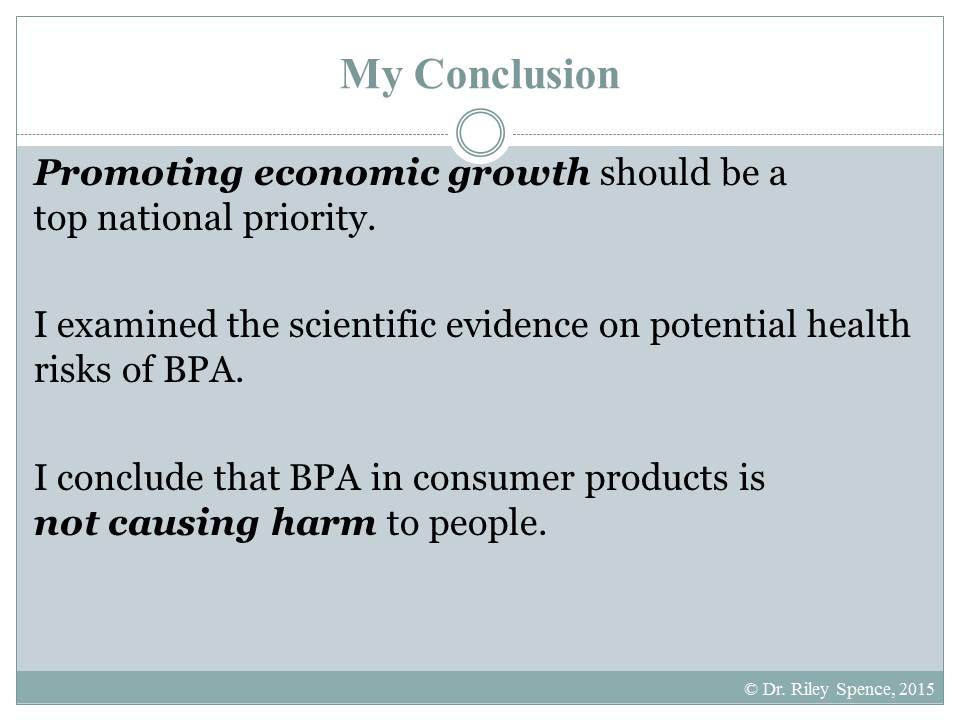


**Part B. Experiment 2 Messages**

Condition 1: scientist does not mention values; scientist recommends regulating BPA more strongly


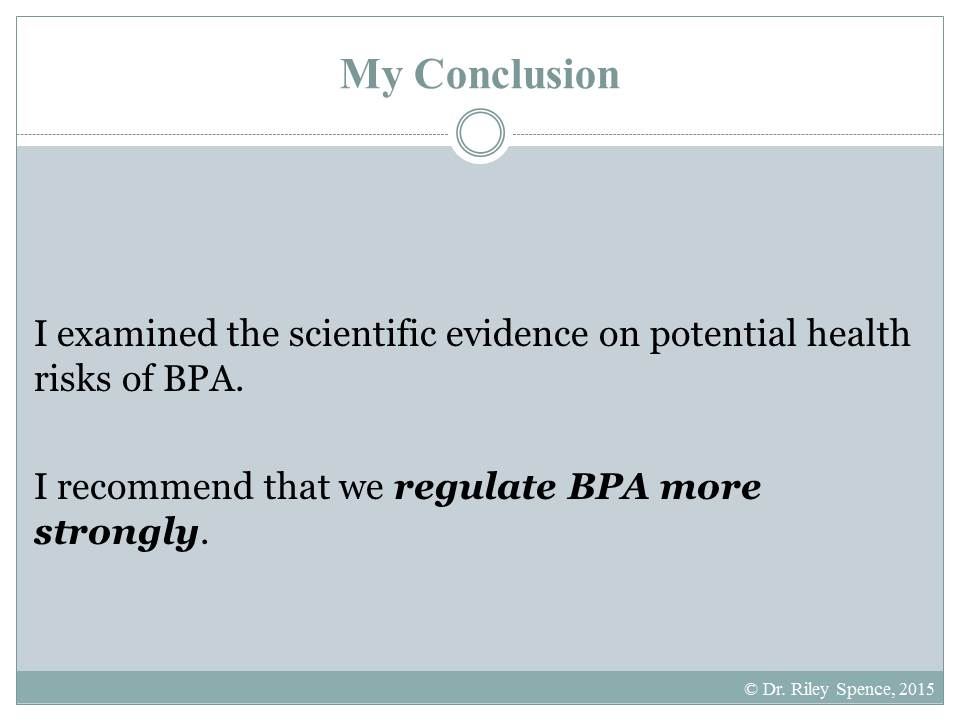


Condition 2: scientist does not mention values; scientist recommends reducing regulation of BPA


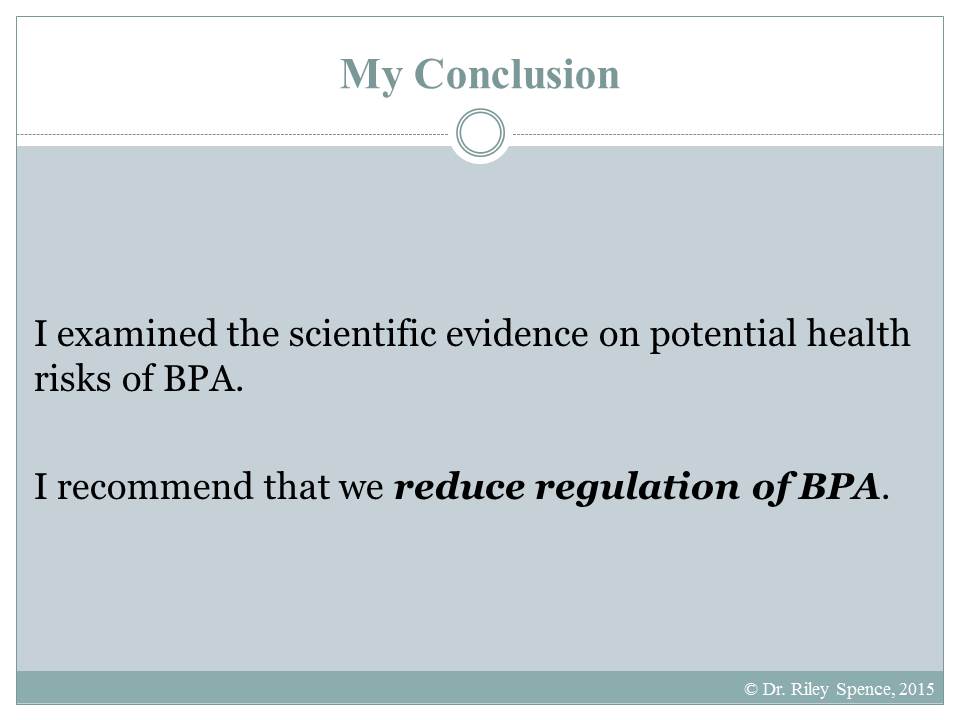


Condition 3: scientist mentions public health values; scientist recommends regulating BPA more strongly


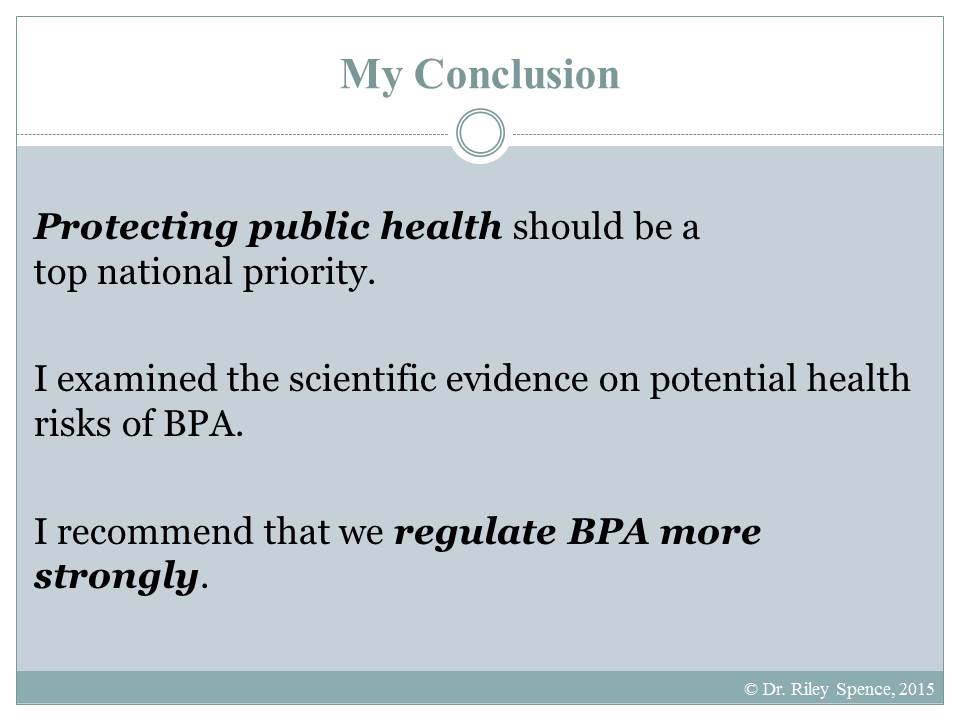


Condition 4: scientist mentions public health values; scientist recommends reducing regulation of BPA


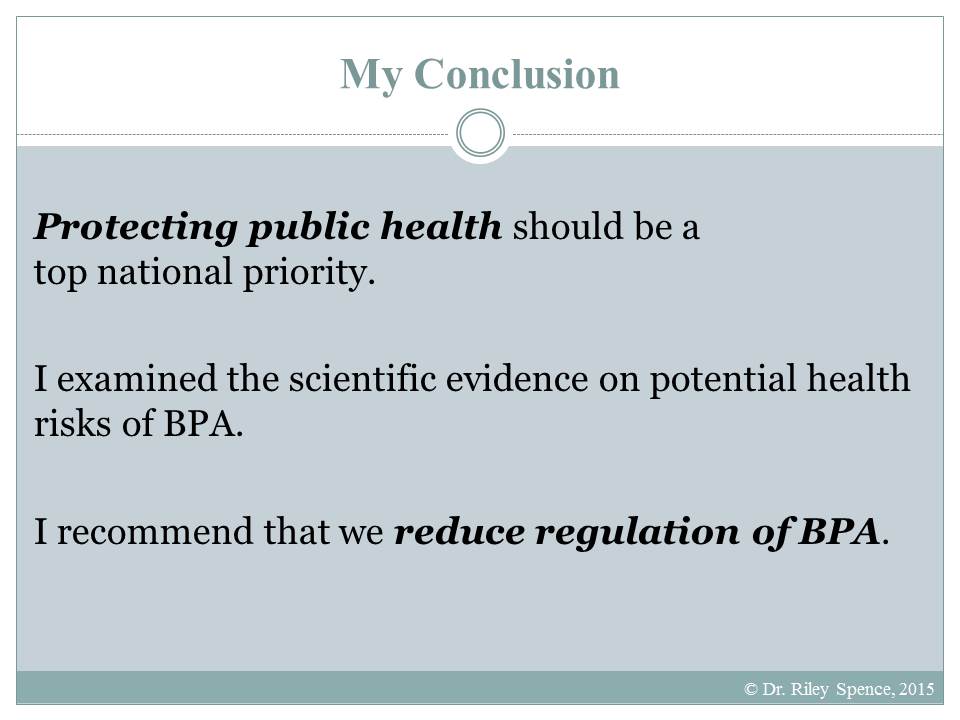


Condition 5: scientist mentions economic growth values; scientist recommends regulating BPA more strongly


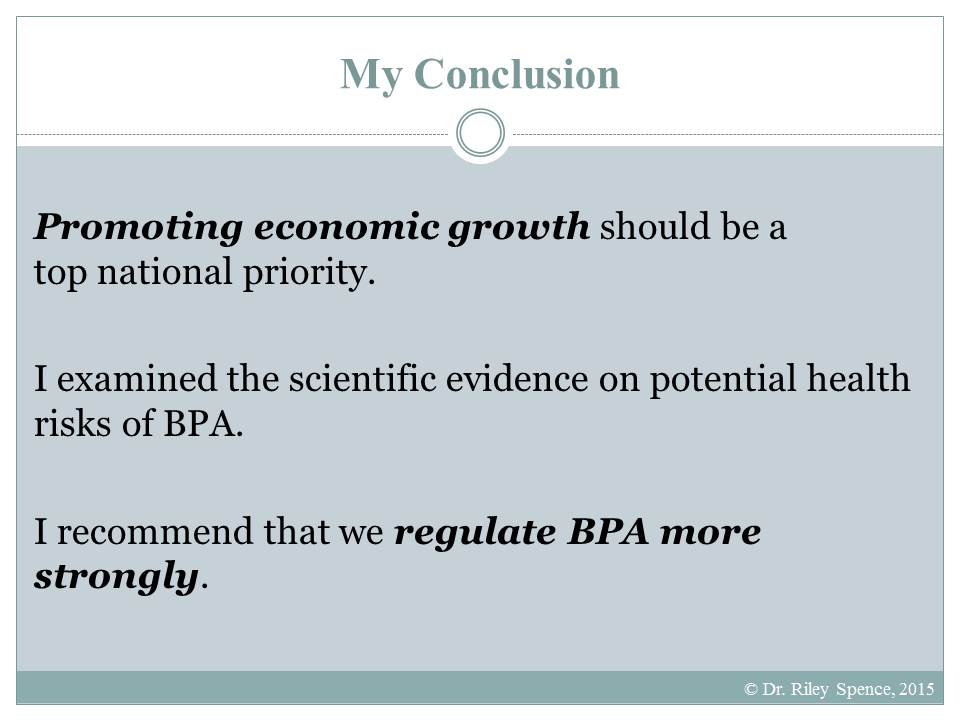


Condition 6: scientist mentions economic growth values; scientist recommends reducing regulation of BPA


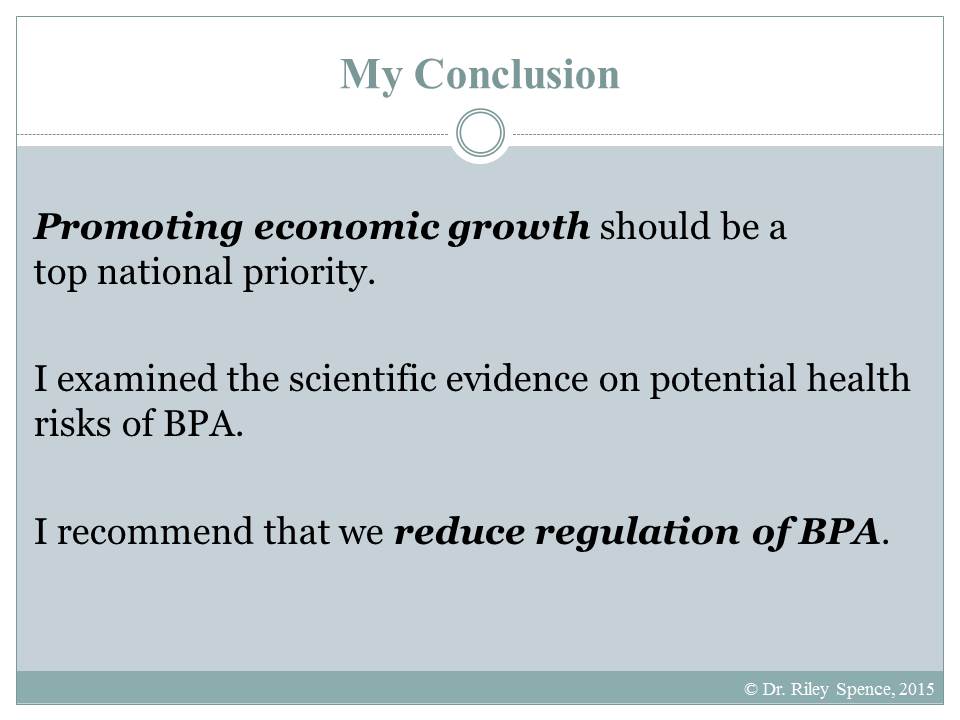

Supplement: S1 File — (DOCX) [file pone.0186049.s001.docx]
